# The Effect of Chinese Square Dance Exercise on Cognitive Function in Older Women With Mild Cognitive Impairment: The Mediating Effect of Mood Status and Quality of Life

**DOI:** 10.3389/fpsyt.2021.711079

**Published:** 2021-07-09

**Authors:** Jindong Chang, Wenbing Zhu, Jia Zhang, Liming Yong, Ming Yang, Jibing Wang, Jiagao Yan

**Affiliations:** ^1^School of Physical Education, Southwest University, Chongqing, China; ^2^Institute of Motor Quotient, Southwest University, Chongqing, China; ^3^College of Physical Education and Health Science, Chongqing Normal University, Chongqing, China; ^4^Qingdao Mental Health Center, Qingdao University, Qingdao, China; ^5^International College of Football, Tongji University, Shanghai, China; ^6^Editorial Department, the Journal of Shandong Sports University, Shandong Sports University, Jinan, China

**Keywords:** cognition, depression, mild cognitive impairment, Chinese square dance, mediation model

## Abstract

The present study aimed to assess the effects of square dance exercise on the cognitive function and quality of life in older women with mild cognitive impairment and to investigate the mediating role of a depressed mood and reduced quality of life in the relationship between square dance exercise and cognition. The study design was a single-blind whole-group controlled trial. A total of 136 eligible participants were divided according to their nursing home into either an experimental or control group. The nursing home grouping was determined by the drawing of lots. The Montreal Cognitive Assessment (MoCA), Quality of Life (SF-12) and Geriatric Depression Scale (GDS-15) were used to assess participants at baseline, week 9, and week 18, respectively. Generalized estimating equations (GEE) were used to compare the results at baseline with mid-test and post-test changes in cognitive function and quality of life. Maximum likelihood estimation (ML) and robust standard errors were used to perform the mediation model. The study results indicated that the experimental group (compared to the control group) had a significant improvement in cognitive function, quality of life, and mood state at baseline in the mid-test and post-test results. The results of this 18-week experiment showed that the exercise–cognition relationship was significantly mediated by a reduction in depressive symptoms (indirect effect: *β* = −0.375; 95% CI = −0.864 to −0.069) and an improvement in quality of life (indirect effect: *β* = −0.678; 95% CI = −1.222 to −0.290). This study revealed the effects of moderate-intensity square dance exercise on cognitive function and quality of life in older Chinese women with mild cognitive impairment and explored the potential mediating mechanisms. These findings can be used to inform the development of public health policies to promote brain health in older adults with mild cognitive impairment.

## Introduction

Global aging has led to an increased focus on the aging process in the field of public health. There is a transition zone in which people move from normal aging to dementia, described by the term mild cognitive impairment (MCI) (1). Individuals with mild cognitive impairment are at a high risk of developing dementia (2). Whereas, the annual conversion rate from mild cognitive impairment to dementia is estimated to be 10–15% (3), the annual prevalence of dementia in the entire elderly population is estimated to be 1–3% (4). Current global estimates of the prevalence of mild cognitive impairment range from 9.6–21.6% (5–7). The high prevalence of mild cognitive impairment leading to dementia emphasizes the need to implement effective therapeutic approaches at this stage, and preventive interventions for this disorder have been widely explored.

Non-pharmacological interventions are still considered to be the main form of treatment for older adults with mild cognitive impairment (2). Among the various non-pharmacological interventions, physical activity has been widely explored as a low-cost, low-risk, and easily accessible lifestyle intervention, and its effectiveness in promoting brain health in elderly care is well known (8). A recent systematic review of randomized controlled trials showed that physical activity, including aerobic and resistance training, is low to moderately effective in promoting cognitive function in older adults with MCI. Sensitivity analyses showed that moderate-intensity aerobic exercise interventions had the best cognitive function effects compared to other exercise interventions (9, 10). The cognitive benefits of physical activity among older adults with mild cognitive impairment in China are still being explored. A 12-week study of a square dance intervention for older adults in one nursing home in northeastern China showed that square dancing is a promising strategy for older adults with mild cognitive impairment and that long-term adherence to square dancing may be beneficial (11). Furthermore, despite the considerable challenges that mild cognitive impairment poses to patients' daily lives, the impact of physical activity on quality of life in this patient population has rarely been studied. The limited evidence related to this phenomenon reports negative results (12, 13), raising questions about whether the cognitive benefits of exercise interventions are transferable to the overall well-being of patients with mild cognitive impairment.

A recently conducted 16-week moderate-intensity aerobic stepping intervention program in Hangzhou, China, reported positive results and identified reduced depressive symptoms and improved sleep quality as part of a potential possible mechanism involved in the exercise–cognition relationship (14). Despite the considerable evidence supporting the role of physical activity in enhancing cognitive function, studies examining the mediating mechanisms behind the exercise–cognition relationship remain scarce. Song et al. (14) identified depressive symptoms and sleep as possible mediating variables in the exercise–cognition relationship. Considering that depressive symptoms are a significant risk factor for predicting cognitive decline (15), and depressive symptoms are strongly associated with quality of life (16). This study will select square dancing as an aerobic exercise intervention to further investigate the role of improving the mood (depression) and quality of life (spiritual) of elderly adults in the exercise–cognition relationship. Since square dancing is popular among older Chinese women, with only a small number of middle-aged and older men taking part in this activity, older women were selected as the participants in this study.

**Figure 1:**
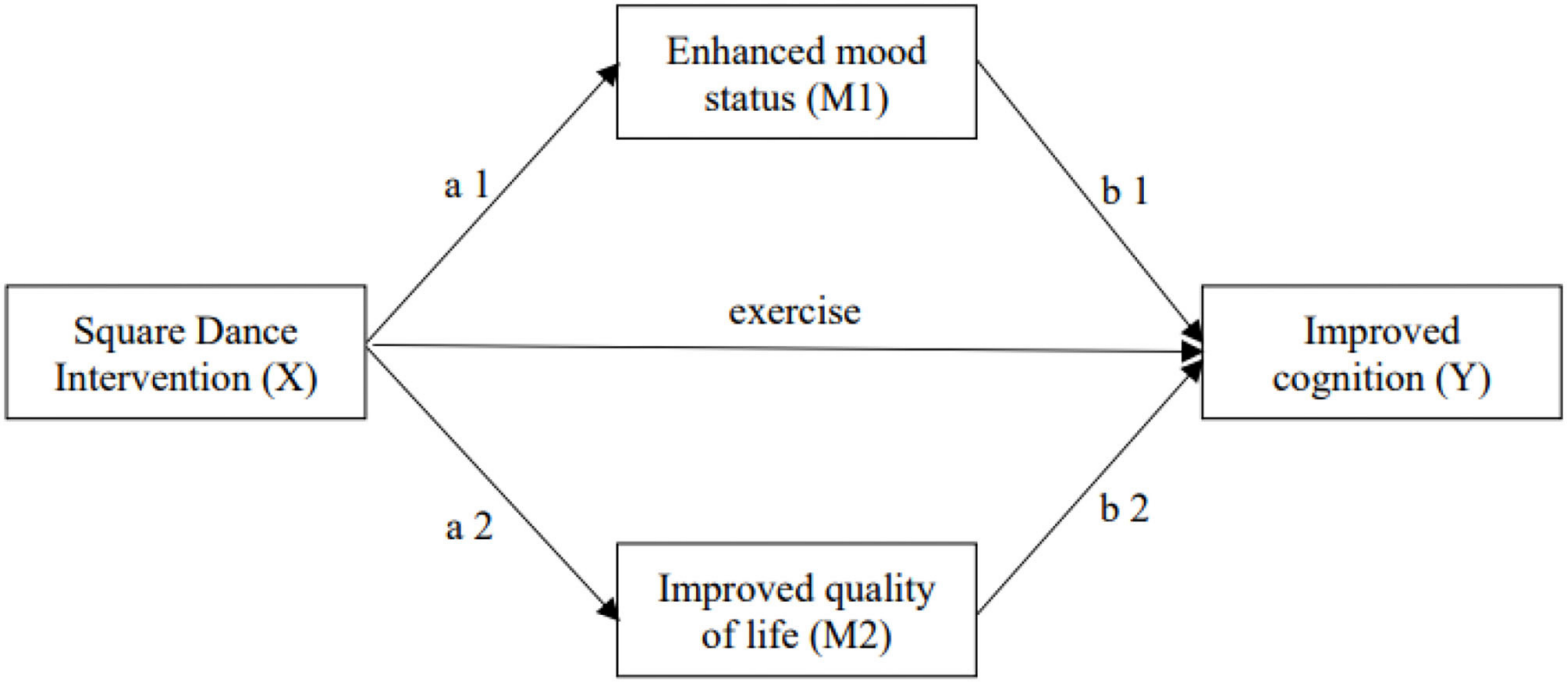
The hypothesis-mediating model.

This study aimed to assess the effects of square dance exercise on cognitive function and quality of life in older women with mild cognitive impairment and to investigate the mediating role of depressed mood and reduced quality of life in the relationship between square dance exercise and cognition. The hypothetical mediator model is presented in Figure 1. The following study hypotheses were tested in older women with mild cognitive impairment.

H1: An 18-week moderate-intensity square dance exercise program will be more effective for improving cognitive function and quality of life in the experimental group than in the control group (i.e., the daily life group).

H2: An 18-week exercise program will be better than a 9-week exercise intervention program in terms of improving cognitive function and quality of life.

H3: An 18-week square dance exercise program will have an effect on cognitive function, improving depressive symptoms and quality of life.

## Methods

### Study Design

This study was a single-blind whole-group randomized controlled trial. According to their nursing home, the eligible participants were randomly enrolled in either the Chinese square dance exercise program or the normal life group. The random sequence was generated by the drawing of lots. Two nursing homes served as the experimental group (EG), and two other nursing homes served as the control group (CG). Data collection was completed by two research assistants who were not involved in the square dance training. Outcomes and mediating variables were measured by face-to-face interviews at baseline before randomization grouping, at the mid-study intervention (week 9), and at one week after completion (week 18). Single blinding was maintained because the research assistants involved in the post-test data collection did not have any information on the subgroup assignment.

### Participants

The sample size required for the experiment was calculated using G^*^Power software. Independent sample *t*-tests were used to analyze the results of the baseline and post-experimental changes. Based on the effect size (Cohen's d = 0.58), 80% power, and a 5% level I error, we estimated that a minimum of 48 participants were required per group. We recruited 10% more subjects than required to account for potential participant dropouts; therefore, a minimum of 53 participants per group were recruited. Participants were recruited from four nursing homes in Chongqing as long as they met the following criteria: (1) at least 60 years old; (2) presented with a subjective cognitive decline in the previous year; (3) obtained a score of <26 on the Montreal Cognitive Assessment (MoCA) scale (plus an extra 1 point if they had achieved 12 years of schooling); (4) attained a score of <26 on the Ability for Daily Living (ADL) assessment. Participants were excluded if they were: (1) taking medication for cognitive impairment; (2) had a neurological disorder (e.g., Parkinson's disease, stroke, multiple sclerosis); (3) had an acute or chronic condition that prevented exercise; (4) performed regular exercise (≥30 min/day, ≥3 days/week) within the past 6 months or had sustained exercise experience for more than 5 years (16). All participants signed a written informed consent form before the study began.

### Intervention

Chinese square dance is a popular form of aerobic exercise for middle-aged and older women. The dance has a simple, easy-to-learn structure and is suitable for older individuals to use as a form of exercise. We chose Chinese square dance as the exercise approach for the experimental group. Square dance music has a simple melody, low movement activity is required, and the central movement structures are handclapping, high-fiving, chest expansion, arm extension, and leg kicking (https://www.youtube.com/watch?v=AYEasAhzHI0) (16).

One week before starting the experiment, two national social sports instructors with professional dance training gave three lessons in two nursing homes to ensure that each participant had mastered the basic movements. After 1 week of study, we provided square dance instructional videos for the experimental group to review. During the teaching process, one instructor demonstrates in front of the participants, and another instructor assists in teaching and correcting incorrect movements. For the formal experiment, an instructor is responsible for leading the practice, and the nursing home arranges a staff member to assist with teaching and to ensure the safety of participants.

The intervention group performed the square dance exercise program outdoors three times a week for 30 min each (during periods of inclement weather, square dance exercise was held indoors). The exercise was led by a professional national social sports instructor on Monday, Wednesday, and Friday evenings from 7:00 p.m. There was a 5-min warm-up exercise (joint finger movement, etc.) before the official start, followed by a 30-min square dance exercise and a 5-min relaxation exercise (i.e., deep breathing and stretching) at the end of the session. During the square dance exercise, participants were required to wear a sports watch to monitor their heart rate. Participants' heart rates during exercise were controlled at 100–140 beats per minute. The control group was not scheduled for specialized physical activity, but did not restrict their voluntary physical activity.

### Measurements

All outcomes were assessed at baseline, midterm (week 9), and within 1 week of the completion of the trial (week 18) by trained assessors whose group assignments to participants were confidential. The primary outcome measures included participants' demographic characteristics, cognitive function, quality of life, and mood state. All scales used were in the Chinese language, and all test tools were supported by proven reliability and evidence of validity.

The Montreal Cognitive Assessment (MoCA) was used for MCI screening in older adults. The MoCA test includes attention and concentration, executive function, memory, language, visual–structural skills, abstract thinking, and computational and orientational skills, with a total scale score of 30, with a normal result considered to be any score ≥26 (17). Similarly, the scale had a retest reliability of 0.85 (18) and an internal consistency (Cronbach's alpha) of 0.84 (19), as well as good content, concurrent, and construct validity (*p* < 0.01) (19). In addition, the MoCA is relatively brief compared to other complex neuropsychological combinations and was, therefore, more appropriate for older adults with mild cognitive impairment.

The Short-Form 12 Health Survey (SF-12) is a self-reported outcome measure that assesses the effect of health on an individual's daily life. It was used to assess quality of life in this study. The SF-12 is a shortened version of the SF-36, which itself evolved from the Medical Outcomes Study. The SF-12 uses the same eight domains as the SF-36, and the correlation coefficient between both is high (20). The SF-12 includes the assessment of vitality, social functioning, emotions, mental health, general health, physical functioning, role physical, and bodily pain. The first four indicators are used to assess the mental component summary (MCS), while the last four are used to assess the physical component summary (PCS). The SF-12 has good internal consistency, with a Cronbach's coefficient of 0.78. It also has good criterion validity (*p* < 0.001). Higher SF-12 scores indicate a better quality of life (21).

The Geriatric Depression Scale (GDS-15) was used to assess participants' depressive symptoms. GDS-15 comprises 15 items. Participants complete the test by answering “yes” or “no”. The GDS has a total score of 15, with higher scores indicating more depressive symptoms. The Chinese version of the GDS-15 has a retest reliability of 0.728 and a Cronbach's alpha of 0.793. In addition, the scale has good discriminant validity (*p* < 0.001) (22).

### Statistical Analysis

Statistical analyses were performed using SPSS 24.0 (SPSS Inc., USA) and Mplus8.3 software. Descriptive statistics were used to analyze the basic characteristics of the participants. Skewness and kurtosis statistics were conducted to screen for the normal distribution of all continuous variables. The *x*^2^ test and *t*-test were used to examine whether demographic and outcome variables were significantly different between the two study groups at baseline. Generalized estimating equation (GEE) models were performed to investigate changes in outcome variables for the two study groups at pre-and post-test (i.e., group^*^time interaction effects). The data analysis was based on the final number of participants completing the experiment. Effect size estimates were calculated for all mean differences using Cohen's d, which relates mean score differences to the combined standard deviation (23).

The maximum likelihood estimation (ML) and bias-corrected bootstrap methods were used to examine the mediating role of depressed mood and quality of life between participation in the square dance intervention and cognitive change. The number of replication samples was set to 1,000. As suggested by Shrout and Bolger (24), a mediating effect was considered significant at the 0.05 level if the 95% confidence interval (CI) (which did not include zero) indicated significant mediation. The mediational model was performed using MPlus 8.3 software (25).

## Results

### Baseline Participant Characteristics

**Figure 2:**
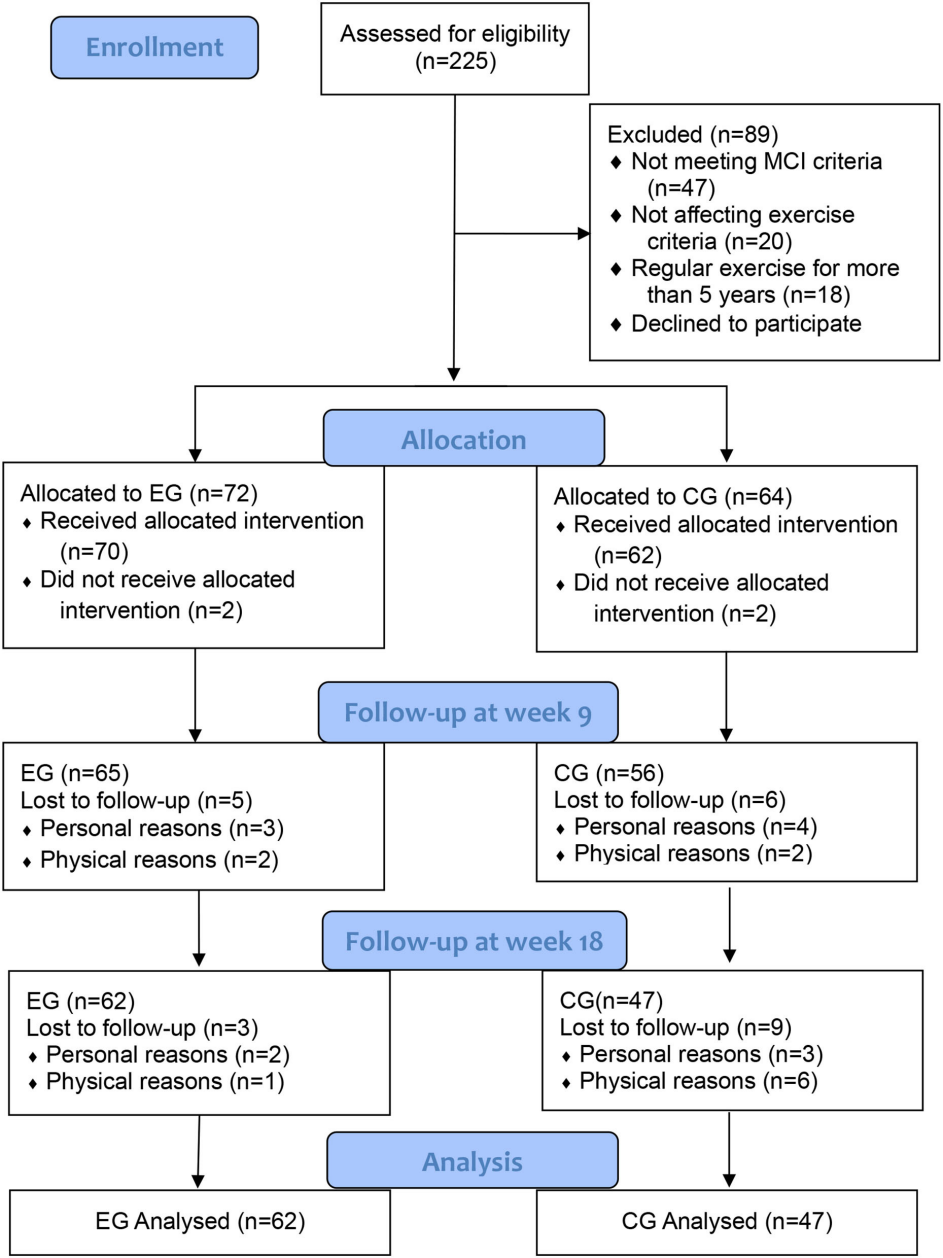
Flowchart of participants recruitment (16).

A total of 225 potential participants were screened for eligibility, of which 136 were enrolled in the study. Figure 2 shows the Consolidated Criteria for Reporting Trials (CONSORT) flowchart, which outlines the participant flow and reasons for dropout throughout the study. By the end of the trial, 27 participants dropped out, resulting in a dropout rate of 19.9%. Ten participants dropped out of the experimental group, with two dropping out before the start, five dropping out before 9 weeks, and three dropping out before 18 weeks. Seventeen participants dropped out of the control group, of which 2 dropped out before the start, 6 dropped out before 9 weeks, and 9 dropped out before 18 weeks. Out of all participants who dropped out, 4 dropped due to disagreement with the subgroup protocol, 11 dropped out for physical reasons, and 12 for personal reasons. The mean age of the participants was 76.29 ± 3.60 and the mean years of education were 8.53 ± 2.06. There were no significant differences in participant demographic characteristics between the experimental and control groups at baseline (Table 1).

**Table 1:** Baseline characteristics of participants.

	**Overall (*n* = 109)**	**EG (*n* = 62)**	**CG (*n* = 47)**	***p***
Age, years	76.29 ± 3.60	76.56 ± 3.60	75.94 ± 3.61	0.369
Education, years	8.53 ± 2.06	8.73 ± 2.05	8.28 ± 2.06	0.261
BMI, kg/m^2^	23.62 ± 2.08	23.63 ± 2.14	23.60 ± 2.03	0.956
MoCA	21.56 ± 2.23	21.61 ± 2.11	21.49 ± 2.39	0.776
GDS	4.92 ± 1.50	4.97 ± 1.41	4.85 ± 1.63	0.690
QLS	90.70 ± 6.74	91.24 ± 6.65	89.98 ± 6.85	0.334
PCS	43.00 ± 6.66	43.09 ± 6.49	42.87 ± 6.95	0.863
MCS	47.70 ± 5.38	48.15 ± 4.97	47.11 ± 5.89	0.320

*Continuous variables are presented as the means ± standard deviations (SDs)*.

*BMI, Body mass index; MoCA, Montreal Cognitive Assessment; GDS, Geriatric Depression Scale; QLS, Quality of Life; PCS, Physical Component Summary; MCS, Mental Component Summary*.

### Experimental Outcome Comparisons

Table 2 lists the baseline, 9-week, and 18-week post-experimental MoCA, GDS, and QLS scores. The MoCA scores showed better improvement in the experimental group than the control group after the 9-week experiment compared to the baseline (*β* = −0.574; 95% CI = −0.948 to −0.200; *p* = 0.003). The effect size was estimated at a moderate level (Cohen's d = 0.59). The MoCA scores showed a significant improvement in the experimental group compared to the control group after the 18-week experiment vs. the baseline (*β* = −0.501; 95% CI = −0.748 to −0.254; *p* < 0.001). The effect size was estimated at a high level (Cohen's d = 0.71).

**Table 2:** Comparison of the experimental and control groups across time.

	**EG (*n* = 62)**	**CG (*n* = 47)**	**Group effect**		**Time effect**		Group*time effect		**Effect size (*d*)**
			***β*(95% CI)**	***p***	***β*(95% CI)**	***p***	***β*(95% CI)**	***p***	
**MoCA**									
Baseline	21.61 ± 2.11	21.49 ± 2.39							
Week 9	22.08 ± 2.03	21.38 ± 2.29	0.451 (−0.584, 1.485)	0.393	1.042 (0.516, 1.568)	<0.001	−0.574 (−0.948, −0.200)	0.003	0.59
Week 18	22.34 ± 1.87	21.21 ± 2.13	0.378 (−0.618, 1.374)	0.457	0.864 (0.428, 1.300)	<0.001	−0.501 (−0.748, −0.254)	<0.001	0.71
**GDS**									
Baseline	4.97 ± 1.41	4.85 ± 1.63							
Week 9	4.55 ± 1.17	4.96 ± 1.38	−0.642 (−1.470, 0.185)	0.128	−0.945 (−1.468, −0.422)	<0.001	0.526 (0.170, 0.881)	0.004	−0.56
Week 18	4.31 ± 1.14	5.02 ± 1.67	−0.532 (−1.207, 0.142)	0.122	−0.746 (−0.996, −0.497)	<0.001	0.416 (0.228, 0.604)	<0.001	−0.88
**QLS**									
Baseline	91.24 ± 6.65	89.98 ± 6.85							
Week 9	93.40 ± 6.19	89.46 ± 6.37	1.406 (−2.023, 4.834)	0.422	4.824 (2.530, 7.118)	<0.001	−2.670 (−4.160, −1.181)	<0.001	0.63
Week 18	94.54 ± 5.63	88.71 ± 6.31	1.019 (−2.074, 4.112)	0.519	3.930 (2.470, 5.389)	<0.001	−2.283 (−3.188, −1.379)	<0.001	0.99
**PCS**									
Baseline	43.09 ± 6.49	42.87 ± 6.95							
Week 9	44.09 ± 6.07	42.88 ± 6.40	0.758 (−2.186, 3.702)	0.614	1.976 (0.735, 3.218)	0.002	−0.982 (−1.800, −0.164)	0.019	0.45
Week 18	44.69 ± 5.35	42.71 ± 6.50	0.654 (−2.205, 3.512)	0.654	1.678 (0.528, 2.828)	0.004	−0.878 (−1.482, −0.274)	0.004	0.43
**MCS**									
Baseline	48.15 ± 4.97	47.11 ± 5.89							
Week 9	49.31 ± 4.40	46.58 ± 5.34	0.648 (−2.410, 3.706)	0.678	2.847 (0.665, 5.030)	0.011	−1.688 (−3.147, −0.229)	0.023	0.44
Week 18	49.84 ± 4.86	45.99 ± 6.41	0.365 (−2.119, 2.849)	0.773	2.252 (0.910, 3.594)	0.001	−1.405 (−2.245, −0.566)	0.001	0.63

Participants in the experimental group showed a better improvement in depressed mood state scores after 9 weeks and at baseline compared to the control group (*β* = 0.526; 95% CI = 0.170 to 0.881; *p* = 0.004). The effect size for the change in the overall GDS score was estimated to be moderate, with Cohen's d = −0.56. Participants in the experimental group were found to have significantly improved depressed mood state scores after 18 weeks and at baseline compared to the control group (*β* = 0.416; 95% CI = 0.228 to 0.604; *p* < 0.001). The effect size for the change in the overall GDS score was estimated to be large, with Cohen's d = −0.88.

Participants in the experimental group showed significant improvements in quality of life scores at baseline and after 9 weeks compared with controls (*β* = −2.670; 95% CI = −4.160 to −1.181; *p* < 0.001), with a moderate effect size estimate (Cohen's d = 0.63). Participants in the experimental group showed significant improvements in quality of life scores at baseline and after 9 weeks compared with controls (*β* = −2.283; 95% CI = −3.188 to −1.379; *p* < 0.001), with a large effect size estimate (Cohen's d = 0.99).

### Results of the Mediating Analysis

**Figure 3:**
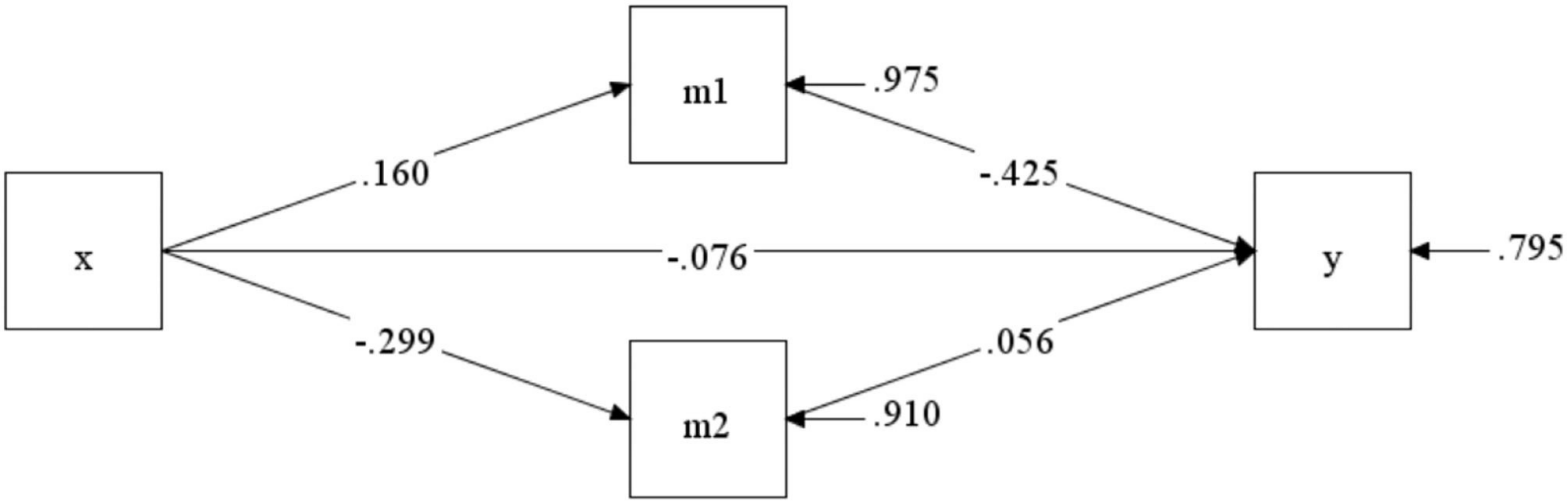
The mediating model after 9-week intervention.

The GEE results of the post-test after 9 weeks of the program indicated that the experimental group had significant improvements in depressed mood states (*β* = 0.526; 95% CI = 0.170 to 0.881) and quality of life (*β* = −2.670; 95% CI= −4.160 to −1.181) compared to the control group. The two mediating variables showed differential changes, supporting the further exploration of the mediating effects. Figure 3 describes the mediation model for the 9-week square dance exercise intervention. The indirect effect of square dance exercise on cognitive function via an improvement in depressed mood (*β* = −0.294; 95% CI = −0.828 to 0.046) and quality of life (*β* = −0.073; 95% CI = −0.443 to 0.134) was not significant.

**Figure 4:**
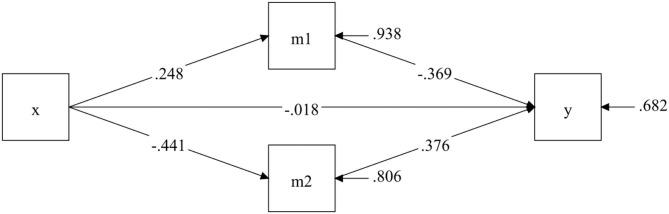
The mediating model after 18-week intervention.

The GEE results of the post-test after 18 weeks of the program indicated that the experimental group had significant improvements in depressed mood states (*β* = 0.416; 95% CI = 0.228 to 0.604) and quality of life (*β* = −2.283; 95% CI = −3.188 to −1.379) compared to the control group. The significant differences in the characteristics of the two mediating variables support further exploration of their mediating roles. Figure 4 depicts the mediation model for the 18-week square dance exercise intervention. The square dance intervention had a significant indirect effect on cognitive function, achieved by an improvement in depressive symptoms (*β* = −0.375; 95% CI = −0.864 to −0.069) and quality of life (*β* = −0.678; 95% CI = −1.222 to −0.290).

## Discussion

The present study aimed to examine the effects of 18 weeks of moderate-intensity square dance exercise on cognitive function and quality of life in older women with mild cognitive impairment and to investigate the mediating role of a depressed mood and reduced quality of life in the relationship between square dance exercise and cognition. This study found that cognitive function deteriorated over time in the control group, while participants with regular participation in square dance exercise showed significant improvements in the same health parameters. This finding was consistent with the findings of Song et al. (14), which suggested that moderate-intensity aerobic exercise may serve the purpose of preventing disease deterioration in older adults with mild cognitive impairment. The mediation analysis further confirmed that the cognitive benefits of square dance exercise were demonstrated by its positive effects on two cognitive risk factors, which included depressed mood and poor quality of life.

The cognitive benefits of an 18-week period of square dance exercise are consistent with the results of previous studies in older adults with mild cognitive impairment. However, some previous studies have shown that at least 6 months of exercise is required to produce such beneficial effects (12, 26–28), while some studies have shown that 12 or 16 weeks can produce such beneficial effects (11, 14). The present study was conducted using two tests, at 9 and 18 weeks, to test whether both protocols achieved similar effects. The results of the study showed that both 9 and 18 weeks produced positive benefits, but the effect size change was greater for the 18-week intervention than the 9-week intervention. This difference can be explained in terms of the study design protocol. First, ACSM recommends an exercise program of 16 weeks or longer, with regularity. The 18-week, three-times-a-week regular exercise program utilized in this study is consistent with the ACSM guideline recommendations. Previous studies have noted that regular aerobic exercise (i.e., up to twice per week) results in cardiovascular and mood-related changes, which benefit cognitive function (27, 28). Second, the use of a sports watch to monitor participants' exercise heart rates meant that we were able to establish that square dancing provided a moderate level of aerobic exercise intensity. The exercise intensity was therefore controlled and may have been more conducive to achieving positive exercise outcomes. Exercise intensity monitoring has also only been utilized in another recent study (14), despite the fact that it has been rarely addressed in previous studies (27, 28). Third, the use of square dance exercises rather than regular fitness activities such as walking, which provide increased physical movement and musical rhythms, is an arrangement that may require participants to follow instructions successfully throughout the workout, thus inadvertently providing a form of attention and short-term memory training. Moderate results were achieved in terms of cognitive function in older women with mild cognitive impairment after 9 weeks of square dance exercise; however, even better results were achieved in terms of the cognitive function improvement after 18 weeks of exercise. The comparison of the mid-experimental and post-experimental results allows us to better explain the exercise intervention benefits that can be obtained through regular and consistent exercise.

The present study identified the benefits of square dance exercise in mitigating the effects of cognitive decline on quality of life in older adults with mild cognitive impairment. The use of two test design protocols for the current exercise program may explain the different findings compared to previous studies in which similar effects were produced following 12 weeks of square dance exercise (woof) or a 6-month moderate-intensity aerobic walking program (13, 16). Participants in the current study showed significant improvements in PCS. These improvements in cognitive function may have a positive impact on a person's perception of health. The present study further validates the previous literature, which suggests that cognitive decline in individuals with mild cognitive impairment disrupts their activity patterns, reduces their social engagement, and leads to clients feeling less empowered and having a poor self-concept (29, 30). The present study suggests that regular square dance exercises may help mitigate such harmful effects in patients.

This trial confirmed that the cognition-enhancing impact of square dance intervention was mediated by a reduction in depressed mood and improved quality of life. The findings illustrate the intervention effects of square dance exercise on cognitive function and propose potentially psychologically relevant mediating pathways to explain the relationship between exercise and cognition. These findings are of substantial value because depression and poor quality of life are highly prevalent in older individuals with mild cognitive impairment. In terms of depressive symptoms, the literature reports a 31.8% prevalence of depressive symptoms in the mild cognitive impairment group compared to 5.5% in cognitively healthy controls. (8). In terms of quality of life, a study by Chandler et al. reported a more significant effect of exercise (e.g., yoga) on memory-related quality of life compared to the support group (effect size = 0.43; 95% CI = 0.13 to 0.72) (31). The Nuzum et al. study also confirmed that this effect was strongly associated with quality of life (*r* = −0.38, *p* < 0.01) (32). As confirmed by large-scale longitudinal studies, both depressed mood and poor quality of life can disrupt the normal lives of older adults with mild cognitive impairment and may further contribute to their cognitive decline (33–35).

The mediating variables in the exercise–cognition relationship—depressed mood and quality of life—can be used to explain how exercise mitigates the adverse effects of these two factors on cognitive function from both functional and physiological perspectives. From a functional perspective, depressed mood and poor mental health due to a reduced quality of life are essentially the same. Both conditions are characterized by physical and mental exhaustion and low motivation. These problems can make it difficult for older adults with mild cognitive impairment to concentrate and can cause their cognitive performance to suffer (36). At the same time, improving depressed mood and quality of life through exercise will reduce their dysfunctional effects on cognitive function. From a physiological perspective, depressive symptoms and poor mental health-related quality of life are associated with neurophysiological changes. Depression down regulates neurotrophic factor expression in the brain environment, leading to neuronal atrophy and hippocampal atrophy (37). Therefore, depressive symptoms can be alleviated by regular moderate-intensity aerobic exercise, which can restore neurotrophic factor expression and promote hippocampal neurogenesis (38). Thus, square dance exercise can create favorable functional and physiological conditions and improve cognitive performance in older women with a mild cognitive impairment through its positive effects on depressed mood and quality of life.

## Limitations

This study has several limitations. Firstly, the sample population was selected from older women only. There was no male sample, so it is not representative of the characteristics of the entire group of patients who experience mild cognitive impairment. Secondly, the randomized sample was underrepresented due to the fact that nursing homes were used as the group unit, limiting the generalization of the overall results. Thirdly, 19.9% of the participants dropped out of the study, which may undermine the explanatory power of the findings. Fourthly, there was no evidence to suggest that changes in scoring outcomes implied significant differences in clinical treatment; therefore, the results have limited explanatory power in terms of their clinical significance. Lastly, the square dance exercise intervention program in this study was implemented for only 18 weeks. Therefore, we are unable to provide any conclusions regarding the effectiveness of the intervention over a more extended period. Future research will explore the above-described gaps in more detail.

## Conclusion

This study of 18 weeks of moderate-intensity square dance exercise showed that its effect on improving cognitive function and health-related quality of life in older women with mild cognitive impairment was significant. The mediation model indicated that reducing depressive symptoms and improving quality of life are potential possible mechanisms affecting the square dance exercise and cognition relationship. The evidence of the effectiveness and feasibility of square dance exercise interventions also promotes the need to increase their application in order to prevent further cognitive decline in older adults with mild cognitive impairment.

## Data Availability Statement

The raw data supporting the conclusions of this article will be made available by the authors, without undue reservation.

## Ethics Statement

The studies involving human participants were reviewed and approved by the Scientific and Ethics Committee of Institute of Motor Quotient, Southwest University (IRB NO. SWUIMQ20180109). The patients/participants provided their written informed consent to participate in this study.

## Author Contributions

JC, WZ, LY, and MY: data collection. JC, JZ, LY, and JY: data analysis, conception, and design. JC, WZ, JW, and JY: research design and writing the manuscript and revision. All authors contributed to the article and approved the submitted version.

## Conflict of Interest

The authors declare that the research was conducted in the absence of any commercial or financial relationships that could be construed as a potential conflict of interest.
